# Is Autophagy Always a Barrier to Cisplatin Therapy?

**DOI:** 10.3390/biom12030463

**Published:** 2022-03-17

**Authors:** Jingwen Xu, David A. Gewirtz

**Affiliations:** 1 School of Pharmacy, Guangdong Pharmaceutical University, Guangzhou 510006, China; jingwen_xu@gdpu.edu.cn; 2 Department of Pharmacology and Toxicology, Virginia Commonwealth University, Massey Cancer Center, 401 College St., Richmond, VA 23298, USA

**Keywords:** autophagy, chloroquine, cisplatin, p53, resistant

## Abstract

Cisplatin has long been a first-line chemotherapeutic agent in the treatment of cancer, largely for solid tumors. During the course of the past two decades, autophagy has been identified in response to cancer treatments and almost uniformly detected in studies involving cisplatin. There has been increasing recognition of autophagy as a critical factor affecting tumor cell death and tumor chemoresistance. In this review and commentary, we introduce four mechanisms of resistance to cisplatin followed by a discussion of the factors that affect the role of autophagy in cisplatin-sensitive and resistant cells and explore the two-sided outcomes that occur when autophagy inhibitors are combined with cisplatin. Our goal is to analyze the potential for the combinatorial use of cisplatin and autophagy inhibitors in the clinic.

## 1. Introduction

Cisplatin (cis-diamminedichloroplatinum (II)) was first approved for clinical use by the FDA in 1978 and has continued to be used as a first-line chemotherapeutic agent for the treatment of approximately 50% of solid tumors, including lung, head and neck, breast, testicular, ovarian, prostate, and bladder cancers [1,2]. Cisplatin was initially synthesized by Michel Peyrone in 1845 and therefore was initially called Peyrone’s salt. In testing the effects of platinum compounds on *E. coli* proliferation, Rosenberg and his group found that cisplatin also has inhibitory effects on sarcoma 180 and L1210 leukemia cells [3,4]. Prior to this time, chemotherapeutic drugs in the clinic were all natural or synthetic organic compounds, and cisplatin became the first antitumor candidate containing heavy metal elements. After approximately 15 years of preclinical experimentation, through 1975, clinical trials led by the J.M. Hill laboratory confirmed the antiproliferative activity of cisplatin against multiple solid tumors [5]. Although cisplatin has a wide range of antitumor activity, its side effects continue to limit its therapeutic use and efficacy. In the clinic, patients treated with cisplatin often experience symptoms of renal tubular necrosis (nephrotoxicity), hearing loss or cochlear damage (ototoxicity), and peripheral sensory neuropathy (neurotoxicity) [6]. These side effects appear more frequently with increasing doses of the drug. In addition to side effects, patients with solid tumors will frequently develop resistance to cisplatin, forcing physicians to consider other treatment options. As cisplatin resistance is often associated with cross-resistance to other commonly used cytotoxic chemotherapeutic drugs, such as doxorubicin and etoposide, this results in a reduction in treatment options [7]. There are many factors leading to cisplatin resistance, including alterations in DNA metabolism, epigenetic and transcriptional modifications, activation of drug efflux systems, and subcellular drug localization and translocation [8].

The mechanisms mediating the antitumor actions of cisplatin have been studied for decades, with DNA being the primary drug target. Once inside the cell, cisplatin undergoes aquation to form [Pt(NH3)2Cl(OH2)]+ and reacts with DNA to form monoadducts, interstrand, intrastrand or DNA–protein cross-links, affecting the DNA double helix structure and nucleosomes of cancer cells [9,10]. This leads to replication and transcriptional repression, and DNA double-strand breaks (DSBs), which then initiate DNA repair. Once DNA repair fails or is overwhelmed by excessive DNA damage, cell death is triggered [11]. An increased capacity to repair DNA is considered as the most significant feature of platinum-resistant cells [12,13].

The central downstream event following cisplatin interaction with cellular DNA is apoptosis [14,15,16]. The intrinsic pathway of cisplatin-induced apoptosis involves the promotion of oxidative stress, whereby cisplatin-treated cells accumulate excessive reactive oxygen species (ROS) (hydroxyl radicals and superoxide). Abnormally accumulated ROS damages mitochondrial respiratory function, leading to mitochondrial dysfunction [17]. ROS, influencing the pro-apoptotic protein Bax, also cause damage to mitochondrial DNA and a reduction in mitochondrial membrane potential, which promotes mitochondrial destruction. Cytochrome c and caspase 9 are then released by damaged mitochondria and evoke a cascade of caspase cleavage reactions [18].

The extrinsic pathway of cisplatin-induced apoptosis is mediated via a type-II membrane protein that activates the Fas receptor in conjunction with the Fas ligand, thereby promoting the formation of the apoptosome complex by the Fas-associated death domain and pro-caspase 8. This apoptosome complex activates caspase 3, caspase 6, and caspase 7, ultimately leading to apoptosis [19]. In addition, cisplatin generally arrests cells in the G1/S or G2 phase of the cell cycle, providing time for repair of damaged DNA prior to DNA synthesis. When cells fail to repair DNA damage at the cell cycle checkpoints, they are forced to re-enter the cycle prematurely, progressing to apoptosis [20,21]. As a “gatekeeper”, the activation of p53 also contributes to cisplatin-induced tumor cell apoptosis [22]. In addition, the p21, MDM2, GADD45 [23], MAPK pathway [24], and PI3K/Akt pathways [25], which are related to p53 and cell cycle regulation, have all been shown to be involved in cisplatin-induced apoptosis.

Macroautophagy (which we will refer to as autophagy) is a critical process in eukaryotic cells whereby superfluous organelles, misfolded proteins, and other cellular debris are cleared, restoring a state of cellular equilibrium [26]. This process is an evolutionarily conserved process whereby cellular debris or toxic cellular components are engulfed by the autophagosome, a double-layered membrane structure, and transported to acidic lysosomes, where they undergo degradation and recycling [27]. Autophagy occurs in cells under nutrient-poor conditions, responding to the decline in external energy sources. Therefore, autophagy is generally considered to reflect a survival-promoting function. However, if autophagy is continuously or overly activated, cell death will be triggered. Upon cisplatin treatment, autophagy induction has been detected in both cisplatin-sensitive and cisplatin-resistant cancer cells. In fact, the basal level of autophagy was significantly elevated in cisplatin-resistant cells [28,29,30,31].

Defective apoptosis is one cause of cisplatin resistance, which confers a survival advantage to tumor cells. This defect facilitates the generation of cellular stress-mediated autophagy, which precedes or effectively blocks the apoptotic cascade. A large number of studies have shown that when cisplatin-induced autophagy is inhibited in cancer cells, the manner of cell death switches to apoptosis [28]. Therefore, taken together, cisplatin-induced autophagy is often considered one of the primary factors thwarting its chemotherapeutic effects. However, the role of autophagy is often far more complex than has been appreciated.

In addition to autophagy and apoptosis, the tumor cell response following cisplatin treatment can include cellular senescence, as in some cases, persistent DNA damage leads to long-term growth arrest [32]. Although senescence was previously considered an irreversible response after chemotherapy, recent studies from a number of laboratories, including our own, have shown that tumor cells have the capacity to escape from this therapy-induced senescence [33]. In this review, we focus on the influence of cisplatin-induced autophagy on the response of solid tumors to this therapy.

## 2. Cisplatin-Induced Autophagy

### 2.1. Cisplatin Resistance

**Figure 1:**
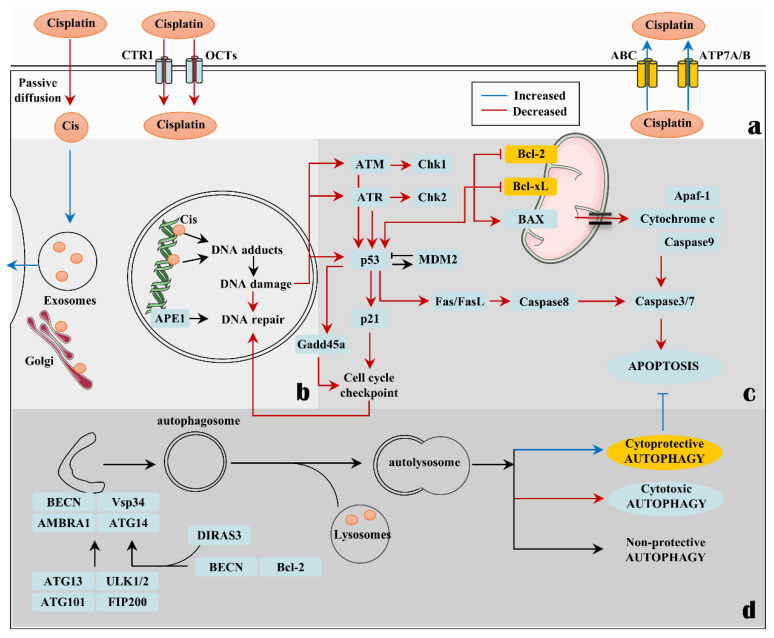
Primary mechanisms of cisplatin resistance. (**a**) Decrease in DNA adduct levels. Inward transport: copper transporter 1 (CTR1) and organic cation transporters (OCTs). Outward transport: ATP-binding cassette (ABC) multidrug transporters (including the multidrug resistance proteins and multidrug resistance-associated protein families). ATP7A/B, which belongs to the copper-transporting P-type ATPase, is the response for delivering copper into the organelles and removing the excess copper out of cells. (**b**) DNA damage recognition defects and increased DNA damage tolerance. (**c**) Inhibition of apoptosis. (**d**) Induction of cytoprotective autophagy. APE1: apurinic/apyrimidinic endonuclease 1; ATM: ataxia telangiectasia mutated protein; ATR: ataxia telangiectasia and RAD3-related protein; AMBRA1: activating molecule in Beclin1-regulated autophagy.

Clinical resistance is defined as the failure of tumor cells to undergo apoptosis at clinically relevant doses or at clinically achievable plasma drug concentrations. The mechanisms conferring cisplatin resistance are numerous and include the following (Figure 1):

**(a) Decrease in DNA adduct generation**: This can result from a number of factors including decreased drug uptake, increased drug efflux, interference with intracellular trafficking and subcellular distribution, increased levels of glutathione (GSH) and the cysteine-rich metallothionein in the cytoplasm in response to cisplatin activation [34], and increased DNA adduct repair by non-homologous recombination. Among these, the most attention in recent years has been paid to the intracellular trafficking and subcellular distribution of cisplatin. Researchers have detected increased cisplatin accumulation in cellular compartments such as Golgi, lysosomes, melanosomes, and exosomes [35], but how cisplatin accumulates in these organelles has not been fully elucidated. Among these, the lysosomal transport of cisplatin [36] was demonstrated to be associated with reduced cytotoxicity (and even resistance) to this drug [37,38,39]. According to this view, lysosomes are regarded as a detoxifying organelle that sequesters both metals and metal-containing drugs and removes them via the exosomal pathway. By doing so, lysosomes tend to reduce the cellular accumulation of cisplatin via exocytosis or its reduced uptake (or both) and transfer the drug to other subcellular targets, thus reducing its cytotoxicity [38,40].

**(b) DNA damage recognition defects and increased DNA damage tolerance**: Damage recognition proteins such as the mismatch repair (MMR) complex generally promote cisplatin-mediated apoptosis after ineffective repair of DNA adducts. However, if the integrity of the MMR complex is lost, the response to cisplatin is significantly reduced [41].

**(c) Inhibition of apoptosis**: As mentioned in the introduction, cisplatin is a potent inducer of apoptosis. A loss of apoptotic signals elevates the threshold of DNA damage for inducing cell death, which is also one of the ways that cells improve DNA damage tolerance [42]. p53 is one of the key factors regulating cisplatin-induced apoptosis, as p53 not only participates in apoptosis but also in the activation of checkpoints following platinum–DNA complexation [43]. Thus, when p53 is mutated, the three-dimensional structure of p53 is altered and can no longer bind to DNA in a sequence-specific manner to transactivate proteins, including cyclin-dependent kinase (CDK) inhibitors, p21Waf1/Cip1, p53 feedback inhibitor murine double minute 2 (MDM2) [44], the growth arrest and DNA damage-inducible Gadd45a gene, and the BCL-2 family, where apoptosis resistance occurs, thereby reducing the susceptibility of tumor cells to cell death [45,46,47].

**(d) Induction of cytoprotective autophagy**: In response to chemotherapy, autophagy can exhibit several functional forms, including a cytoprotective form that plays a pro-survival role, a cytotoxic form that promotes tumor cell death, and a nonprotective form, which does not seem to directly affect cell proliferation or apoptosis [48]. When proposing that autophagy may suppress cisplatin sensitivity or lead to drug resistance, it is necessary to distinguish the function of autophagy. This is because although cisplatin often induces the cytoprotective form of autophagy, whereupon autophagy inhibition enhances cisplatin efficacy, our research group as well as other laboratories have found that cisplatin can also induce non-protective autophagy and cytotoxic autophagy [49,50]. Furthermore, we and others have reported on the existence of an “autophagic switch” due to manipulation of the status of specific genes [51,52,53,54]. For example, after cisplatin treatment, non-protective autophagy in p53 H460 cells can be “switched” to cytoprotective autophagy in CRISPR/cas9 p53 H460 cells [50].

### 2.2. Factors That Affect the Role of Autophagy in Cisplatin-Sensitive Cells

**ATM-CHK2 and ATR-CHK1 pathways**: The DNA damage chemotherapeutic drug response has largely been defined in the context of the ataxia telangiectasia mutated (ATM)-CHK2 and RAD3-related (ATR)-CHK1 pathways. Cell cycle-related proteins downstream of ATM and ATR such as p53, p21, MAPK, AMPK, and PTEN are inextricably linked to the proliferation of tumor cells. Reinhardt et al. found that activation of the ATM/ATR-p38 MAPK-MK2 pathway in p53-deficient cells is required for resistance to DNA-damaging chemotherapeutic agents such as cisplatin [55]. However, we as well as other researchers have found that the deletion of p53, AMPK, or PTEN does not affect the induction of autophagy by cisplatin [50,56], suggesting that there must be other key factors that regulate autophagy in cisplatin-treated cells. A further study by Gomes et al. found that in their 3D-rBM cell model, although the p53 status did not affect cisplatin sensitivity, the inhibition of ATR enhanced cisplatin-induced cell death [57]. A recent study by Chen et al. showed that the ATM-CHK2 axis is associated with cisplatin-induced autophagy via modulation of FOXK proteins, members of the forkhead transcription family. While these can act as transcriptional repressors of autophagy, DNA damage promotes phosphorylation of the FOXK proteins via CHK2, resulting in their being trapped in the cytoplasm and thereby promoting protective autophagy. Furthermore, a cancer-derived FOXK mutation also induced FOXK hyperphosphorylation, exacerbating autophagy and drug resistance. Consequently, the combination of chloroquine (CQ) with cisplatin has a strong growth-inhibitory effect in tumor cells expressing these cancer-derived FOXK mutants [56].

**AMBRA1**: Activating molecule in Beclin1-regulated autophagy (AMBRA1) is an important factor in regulating autophagy and cell proliferation. AMBRA1 promotes autophagy by activating ULK1 and the BECN1-PIK3C3/VPS34 complex [58,59]. Using a genetic knockdown approach, AMBRA1 was found to mediate cisplatin-induced autophagy and chemosensitivity in prostate cancer [60] and cervical cancer [61]. A recent study further clarified the mechanism of AMBRA1 as being related to the tumor cell response to cisplatin. Antonioli et al. found that human papillomavirus (HPV)-negative oropharyngeal squamous cell carcinoma (OPSCC) had higher autophagy levels as well as higher AMBRA1 levels compared with HPV-positive OPSCC, while knockdown of AMBRA1 decreased cisplatin-induced cytoprotective autophagy in HPV-negative OPSCC [62]. These studies suggest that AMBRA1 may serve as a potential target in combination with cisplatin when autophagy plays a protective role.

**Galectin-1:** Galectin-1 is a member of a family of galectins with an affinity for β-galactosides, that is involved in cell adhesion, cell cycle progression, and apoptosis [63]. It is detected in the periphery of tumor cells and is involved in various stages of tumor cell proliferation, in addition to its involvement in the inflammatory response [64]. However, an increasing number of studies have indicated the inconsistent biological functions of galectin-1 in intracellular and extracellular compartments on tumor cells (i.e., extracellular galectin-1 induces anti-proliferative effects via the Ras/MAPK pathway, whereas intracellular galectin-1 exhibits the ability to promote tumor transformation) [65]. Due to the diversity of its biological functions, researchers have been attracted to exploring its effect on the antitumor actions of platinum-based chemotherapy drugs. Chung et al. found that galectin-1 was overexpressed in lung cancer cells and the tissues of lung cancer patients and was associated with Ras, p38 MAPK, ERK, and NF-κB. They also found that knockdown of galectin-1 increased the sensitivity of A549 cells to cisplatin [66]. It has also been reported that galectin-1 knockdown promotes cisplatin sensitivity in other tumor cells, such as bulky squamous cervical cancer [67] and epithelial ovarian cancer [68]. Gao et al. asserted that the inhibitory effect of galectin-1 on cisplatin sensitivity may be related to its induction of autophagy [30]. Further analysis of the relationship between galectin-1 and cisplatin-induced autophagy determined that inhibition of autophagy abolished the resistance to cisplatin conferred by galectin-1 in hepatoma cells [69]. Interestingly, however, we noted that in their studies, silencing of ATG5 or pharmacological inhibition of autophagy by Bafilomycin A pretreatment did not enhance cisplatin-induced cell death in Huh7 cells. Therefore, we suggest that cisplatin likely induced non-protective autophagy in Huh7 cells, while the exogenous addition of galectin-1 not only accelerated hepatocellular carcinoma cell death but also promoted the cisplatin-induced autophagy switch from non-protective autophagy to protective autophagy.

**ARHI**: Bast et al. found that the oncogene ARHI (DIRAS3), a gene downregulated in 60% of cervical cancers [70], is involved in cell proliferation [71,72], migration [73], autophagy, and tumor dormancy [74,75] regulation. Their study suggests that ARHI may also have a regulatory role in the cisplatin-induced autophagic switch. These investigators reported that in a nude mouse xenograft model of ARHI-re-expressing SKOV3 cells, treatment with CQ significantly delayed the regrowth of the dormant tumor cells after withdrawal of ARHI [75]. Complementary findings were reported by Li et al., who observed that overexpression of ARHI in TOV12D and ES2 ovarian cancer cells significantly delayed xenograft growth by inhibiting AKT activity and decreasing Bcl-2 expression, inducing apoptosis and autophagic cell death [76]. Bast et al. subsequently found that autophagy inhibition using CQ or shATG5 had no significant effect on cisplatin-induced colony formation or cell survival in ARHI-negative SKOV-3, Hey, and OVCAR4 cells, suggesting that non-protective autophagy was induced [77]. These results again allude to our previous view that cisplatin-induced autophagy is not always promoting or inhibitory to tumor growth and that there is also “nonsense” autophagy (i.e., a non-protective form of autophagy whose inhibition does not alter sensitivity to the autophagy-promoting stimulus). In contrast, ARHI re-expression enhanced the sensitivity to cisplatin both in vitro and in vivo, and the combined treatment with CQ attenuated the enhanced effect of ARHI on cisplatin chemosensitivity, implying that here, autophagy played a role in promoting cell death in ARHI re-expressed cells (e.g., cytotoxic autophagy) [77]. These results suggest that high expression of ARHI can have a positive role in promoting cisplatin activity against cervical cancer. On the other hand, re-expression of ARHI induced the switch to cytotoxic autophagy from non-protective autophagy, which should suggest caution in that that the use of CQ in combination with cisplatin therapy in patients with high ARHI expression has the potential to generate an undesirable outcome.

**ECRG4**: Esophageal carcinoma-related gene 4 (ECRG4) is a novel candidate tumor suppressor gene that has frequently been found to be inactivated by promoter hypermethylation in different cancer types, including esophageal cancer, prostate cancer, gastric cancer, colorectal carcinoma, and glioma [78,79,80]. ECRG4 is also identified as a paracrine factor-activated microglia, which has effects on the chemotaxis of monocytes and potential as a target for anti-tumor therapy [81]. You et al. showed that ECRG4 overexpression not only promotes cisplatin chemosensitivity but also plays a role in the cisplatin-induced autophagic switch. They found that 3-methyladenine (3-MA) had no effect on the survival of cisplatin-induced nasopharyngeal carcinoma CNE1 cells, but it decreased the chemosensitivity of cisplatin in ECRG4-overexpressing CNE1 cells [82]. This suggests that ECRG4 overexpression leads to the cisplatin-induced autophagic switch from nonprotective autophagy to cytotoxic autophagy. However, studies on the relationship between this gene and autophagy regulation are relatively limited.

**PFKFB3**: Upregulation of glycolytic metabolic pathways is associated with cancer progression [83]. The activity of 6-phosphofructo-2-kinase/fructose-2,6-biphosphatase 3 enzyme (PFKFB3) represents one of the rate-limiting steps in mediating the conversion of fructose-6-phosphate to fructose 2,6-bisphosphate (F-2,6-BP) in the glucose metabolic pathway. PFKFB3 is highly expressed in a variety of tumors [84], such as head and neck squamous cell carcinoma [85], hepatocellular carcinoma [86], breast and colon cancer [87], gastric cancer [88], and ovarian cancer [89]. PFKFB3 inhibition was found to induce B16-F10 tumor vessel normalization, impaired metastasis, and improved cisplatin chemosensitivity [90]. Similar results were reported by Li, who found that PKF15, an inhibitor of PFKFB3, sensitized tumors to cisplatin treatment in a xenograft model [91]. Following this, a recent study showed that PFK158, another PFKFB3 inhibitor, also promotes cisplatin chemosensitivity in endometrial cancer through the induction of cytotoxic autophagy via inhibition of the PI3K/Akt/mTOR pathway [92]. These positive outcomes support the potential combination of inhibitors of PFKFB3 with cisplatin.

### 2.3. Factors That Affect the Role of Autophagy in Cisplatin-Resistant Cells

**p53**: As mentioned above, the status of the tumor suppressor p53, which is generally regarded as a key cellular defense mechanism against cancer, can influence cisplatin sensitivity. Tung et al. demonstrated that wild-type p53 in non-small cell lung cancer (NSCLC) cells inhibits the Nrf2 promoter’s activity to promote cisplatin-induced apoptosis. In contrast, mutant p53 causes apoptosis resistance due to its inability to inhibit the activity of Nrf2, followed by the induction of the anti-apoptotic protein Bcl-2 and increased expression of Bcl-XL [93]. These studies support the necessity for p53 in the process of cisplatin-induced apoptosis in NSCLC cells. Although the promoting effect of wild-type p53 on the efficacy of cisplatin is easy to understand, clinical trials 20 years ago found that this effect and the type of tumor are interrelated, and the results have not always been consistent. Clinical trials have found that for the treatment of cervical cancer, p53 wild-type patients do not respond optimally to cisplatin [94]. More interestingly, for testicular cancer, a tumor that is highly sensitive to cisplatin, almost all of the few refractory patients have been found to harbor wild-type p53, which is not consistent with the commonly accepted view that tumors with p53 mutations are more therapy-resistant [95]. In addition, the Kroemer laboratory found that the regulation of autophagy was associated with the subcellular localization of p53. In short, p53 in the cytoplasm inhibited autophagy, whereas p53 in the nucleus, in contrast, induced autophagy [96]. It is tempting to speculate that the influence of p53 on cisplatin-induced autophagy is also inconsistent. A recent study showed that re-expression of p53 in p53-null SKOV3 cells increased cisplatin-induced autophagy and apoptosis in vivo and in vitro, while the induction of p53 in ERK-active Ras-expressing cells did not further induce autophagy but reversed the cisplatin resistance to sensitivity, indicating that the wild-type p53 status determines the role of autophagy in ovarian cancer chemoresistance [97]. This result is consistent with a study from Rosenfeldt et al., who demonstrated that treatment with the autophagy inhibitor hydroxychloroquine (HCQ) significantly accelerated tumor formation in autophagy-competent mice with oncogenic KRAS but lacking p53 [98]. These results all imply that the status of p53 influences the occurrence and the nature of autophagy. However, the outcomes in cisplatin-sensitive cells are complicated. Studies from the Gewirtz laboratory demonstrated that the status of p53 has no effect on the extent of autophagy but instead influence the nature of the autophagy. H460 p53 wild-type cells exhibited nonprotective autophagy in response to cisplatin treatment, whereas CRSPR/Cas 9 knockout of p53 H460 cells demonstrated cytoprotective autophagy [50]. Similar to the outcomes of this work, Tripathi et al. found that cisplatin induced cytoprotective autophagy in p53 knockdown embryonal carcinoma cells [99]. Maycotte et al., however, found non-protective autophagy in p53-null mouse breast cancer cells (67NR and 4T1 cells) after exposure to cisplatin [100]. Unlike these findings, Gomes et al. found that, whether the breast cancer cells or lung cancer cells responded to cisplatin in traditional two-dimensional (2D) or in 3D reconstitution-based membrane cell culture models, cisplatin-induced autophagy appeared to be independent of the p53 status [57]. These results indicate that p53 clearly may play a key role in cisplatin-induced autophagy, and this is particularly important in cisplatin-resistant cells.

**RASSF1A**: RAS association domain containing family 1A (RASSF1A), a tumor suppressor gene frequently inactivated in human cancers, is phosphorylated on ser131 by ATM following DNA damage, leading to an apoptotic response [101]. Koul et al. showed that promoter hypomethylation of the RASSF1A gene plays a role in cisplatin resistance in male germ cell tumors [102]. Similar results were found in a clinical trial after paclitaxel-carboplatin or gemcitabine-cisplatin treatment, where methylation of RASSF1A negatively impacted the prognosis of early-stage NSCLC [103]. Levesley et al. found that cisplatin induced more extensive apoptosis in RASSF1A-complete pediatric medulloblastoma UW228-3 cells, further identifying the RASSF1A tumor suppressor as a promoter of apoptotic signaling pathways [104]. In addition, cisplatin decreased the ability of ATM to phosphorylate RASSF1A-p.133Ser and to affect p53 activation [101]. Because RASSF1A, similar to p53, is a tumor suppressor gene that is involved in cisplatin-induced apoptosis, the relationship between RASSF1A and autophagy has attracted the interest of researchers. Surprisingly, unlike p53, a recent study suggests that the activation of RASSF1A may activate the Keap1-Nrf2 pathway by regulating microtubule-associated protein 1s (MAP1S), thus activating cytotoxicity autophagy to enhance the chemosensitivity of cisplatin in cisplatin-resistant NSCLC [105]. However, further investigation is required to determine whether the tumor suppressor role of RASSF1A is related to the activation of cytotoxic autophagy.

**APE1**: Apurinic/apyrimidinic endonuclease 1 (APE1) can repair DNA damage and regulate select processes related to cell survival, proliferation, and migration through the base excision repair (BER) pathway. Its regulatory role is inextricably linked to nuclear factor-κB (NF-κB) [106], hypoxia inducible factor 1α (HIF-1α) [107], p53 [108], signal transducer and activator of transcription 3 (STAT3) [109], and nuclear factor (erythroid-derived 2)-like 2 (Nrf-2) [110]. Clinical samples and preclinical studies of cisplatin suggest that APE1 is associated with NSCLC invasiveness, a poor prognosis, and cisplatin resistance [111,112,113,114]. Li et al. demonstrated that APE1 was not only overexpressed in cisplatin-resistant A549 cells but also had a correlation with cisplatin-induced autophagy [115]. This conclusion was further refined by Pan et al., whose experiments showed that cisplatin-induced cytoprotective autophagy in KRASG12S-mutant A549 cells and the combined treatment of CQ with APE1 siRNA increased cisplatin sensitivity [116]. The above studies suggest that APE1 overexpression is often detected in NSCLC and is associated with cisplatin-induced cytoprotective autophagy.

### 2.4. The Non-Coding RNA That Affects the Role of Autophagy in Cisplatin-Treated Cells

Francis Crick’s “central dogma” states that genetic information is transmitted through the DNA–RNA–protein sequence. In this process, however, there are always some genes that are not translated into proteins, which are called non-coding RNAs (ncRNAs). Many ncRNAs have been discovered and shown to be involved in regulating cellular processes and pathways in cancer. Some of these long non-coding RNAs (lncRNAs), circular RNAs (circRNAs), and microRNAs were demonstrated to mediate cisplatin-induced cytoprotective autophagy. Metastasis-associated lung adenocarcinoma transcript 1 (MALAT1) is located on chromosome 11q13 and has been identified to be involved in cancer development and progression [117]. Hu et al. found that CQ enhanced cisplatin-induced apoptosis in vincristine-resistant SGC-7901 cells, indicating that cisplatin-induced resistance in gastric cancer cells is associated with autophagy. They also found that lncRNA MALAT1 enhanced cisplatin-induced cytoprotective autophagy by sequestering mir-23b-3p and increasing the level of ATG12 [118]. lncRNA MALAT1 has also been found to be highly expressed in cisplatin-resistant AGS and HGC-27 gastric cancer cells and promotes autophagy through suppression of the miR-30b/ATG5 and miR-30e/ATG5 axes, thereby reducing cisplatin sensitivity [119,120]. In addition, the regulation of cisplatin-induced autophagy by circRNAs has also been reported. Peng et al. found that circCUL2 regulated cisplatin sensitivity through mir-142-3p/Rock2-mediated autophagy activation in AGS/cisplatin and SGC-7901/cisplatin cell lines [121]. It is interesting to note that miRNA is also reported to be involved in cisplatin-induced protective autophagy, but it is more commonly observed in gastric cancer cells. For example, miR-148a-3p reconstitution in cisplatin-resistant cells inhibits cytoprotective autophagy by suppressing RAB12 expression and mTOR1 activation [122]. MiR-216a-5p overexpression decreased Bcl-2 expression, enhanced Beclin1 expression, and activated cisplatin-induced autophagy [123]. However, more interestingly, for the gastric cancer cell line SGC-7901, the research of Zhu et al. showed that CQ also decreased cisplatin sensitivity and induced cytotoxic autophagy [49]. In contrast to the inconsistent reports in gastric cancer cells, the role of ncRNAs seems to be relatively consistent for cisplatin-induced autophagy in lung cancer cells. For example, LncRNA BLACAT1 [124], miRNA-1 [125], miR-181 [126], miR-223 [127], and miR-425-3p [128], whose overexpression was reported to upregulate cisplatin-induced autophagy, were associated with resistance to cisplatin in lung cancer cells.

There are many other reports relating to the regulation of cisplatin-induced autophagy in tumor cells by different species of ncRNA [129], such as the enhancement of cisplatin-induced autophagy in thyroid cancer cells by overexpression of MicroRNA-125b in vivo and in vitro [130]. Although we will not list all of these studies here, it is important to emphasize that virtually all of these studies are missing the necessary experiments to confirm the role of cisplatin-induced autophagy. Although it has been reported that cisplatin-induced apoptotic cell death was enhanced by the blockade of either siRNA-mediated knockdown of Atg7 genetic expression or a pharmacological inhibitor (3-MA) in NSCLC [131], we and other labs indeed observed that the use of CQ or other autophagy inhibitors did not enhance cisplatin-induced apoptotic cell death (i.e., non-protective autophagy) [50,100] or reduce the cell death rate’s decline (i.e., cytotoxic autophagy) [49]. The current status of the research is that many studies are somewhat handicapped by the preconceived notion that cisplatin-induced autophagy is cytoprotective and related to cisplatin resistance. Therefore, after knockdown or overexpression of ncRNAs, a decreased level of autophagy is observed (usually, only downregulation of LC-I to LC3-II is detected), leading to the possibly incorrect conclusion that the specific ncRNA mediated cisplatin chemoresistance by regulating autophagy.

### 2.5. Other Genes That Affect the Role of Autophagy in Cisplatin-Treated Tumor Cells

In addition to the more widely reported genes listed above, the expression of many other genes has been shown to affect cisplatin chemosensitivity by regulating autophagy. These include BCAT1 [132], protein disulfide isomerase family 6 (PDIA6) [133], serum-and glucocorticoid-inducible kinase 2 (SGK2) [134], inhibitor of DNA binding 1 (ID1) [135], O-6-methylguanine-DNA methyltransferase (MGMT) [136], PDZ-binding kinase (PBK) [137], IGF2R [138], caveolin-1 (Cav-1) [139], HSP90AA1 [140], O-GlcNAc transferase (OGT) [141], neutrophil gelatinase-associated lipocalin (NGAL) [142], and GFRA1/GFRα1 (GDNF family receptor α1) [143]. However, it is necessary to strongly emphasize that when studying the relationship between specific genes and cisplatin-induced autophagy, it is critical to clarify the function of autophagy in the study model; otherwise, any conclusions that might be drawn would be lacking a rigorous experimental foundation.

## 3. The Yin and Yang Faces of Autophagy Inhibition in Cisplatin Therapy

Cisplatin treatment promotes autophagy in both cisplatin-sensitive and cisplatin-resistant cells. Consequently, inhibition of autophagy can be considered a strategy for improving cisplatin chemosensitivity [29,30,31]. This is the positive side, which is what we call Yang. However, as discussed in Section 2, the functional activity of cisplatin-induced autophagy is related to different genetic phenotypes and tumor types as well as the microenvironment of the tumor. In addition, preclinical studies have found that pharmacological autophagy inhibitors are not uniformly effective in enhancing the effectiveness of cisplatin and may also exacerbate the side effects of cisplatin toward normal tissue. This is the negative side, which we call Yin. We will next elaborate on the two elements that autophagy inhibition brings to cisplatin treatment in terms of both therapeutic efficacy and side effects.

### 3.1. A Beneficial Treatment Strategy of Autophagy Inhibition Combined with Cisplatin Is Closely Related to Tumor Types

CQ and HCQ are quinoline derivatives that exhibit a variety of activities and are able to cross cell membranes by passive diffusion. These drugs accumulate and are subsequently protonated in acidic vesicles such as lysosomes. This accumulation leads to a weakened acidic environment, thereby disrupting the endolysosomal system [144]. The fusion of lysosomes and autophagosomes is a critical step for the completion of autophagy, sometimes referred to as autophagic flux. CQ interferes with the fusion process and is therefore considered to be an effective inhibitor of late-stage autophagy [145]. The combination of CQ with cisplatin has been reported to not only increase the chemotherapeutic efficacy of cisplatin-sensitive cancer cells [145] but also to effectively improve the chemosensitivity of cisplatin-resistant cancer cells (Table 1). For example, as listed in Table 1 below, CQ increased cisplatin sensitivity by inhibiting autophagy in cisplatin-resistant A549 (A549/cisplatin) [146], endometrial cancer [147], urothelial carcinoma [148], epithelial ovarian cancer [149], esophageal cancers [29], and neuroblastoma [150]. Apart from the generally recognized autophagy inhibitory effect, recent studies have also reported autophagy-independent activities of CQ against breast cancer [100], as well as increased cisplatin sensitivity to laryngeal tumor cells by promoting repolarizing tumor-associated macrophages from M2 to M1 in vivo and in vitro [151].

The above studies appear to suggest that the combination of CQ and cisplatin is indeed a potential approach to overcoming cisplatin resistance, where this sensitizing effect is largely relevant to specific tumor types. For instance, in patients with cisplatin-resistant oral squamous cell carcinoma, inhibition of autophagy does not seem to be an ideal treatment. A recent study showed that although CQ increased the apoptosis rate of cisplatin-treated SSC-4 cells, it had a very limited effect on the apoptosis of cisplatin-resistant SSC-4 cells (only about a 5–7% increase) [152].

For cells that are inherently sensitive to cisplatin, the outcomes for CQ in combination with cisplatin seem to be more relevant to the tumor types. For example, in cisplatin-sensitive glioblastoma, pediatric medulloblastoma cell lines, atypical teratoma/rhabdomyosarcoma cell lines [153], low ARHI-expressing ovarian cancer SKOV3 cells [77], p53 wild-type lung cancer H460 cells [50] and p53-null mouse breast cancer 67NR and 4T1 cells [100], CQ had no significant effect on the activity of cisplatin (Table 1).

In addition to CQ, Bafilomycin-A1 and PI3K inhibitors (3-MA and wortmannin) are also common inhibitors of autophagy and have been reported to increase the chemosensitivity of cisplatin in different tumor cells in a large number of studies, which have been summarized in many reviews [28]. Nevertheless, there are also exceptions. An example is that the 3-MA showed no effect on the cell proliferation rate in cisplatin-treated nasopharyngeal carcinoma CNE1 cells [82]. More interesting examples are cisplatin-resistant gastric cancer KATO-III cells, for which studies have shown that the resistance is independent of MRP1 and MDR1 and rather linked to Aldoketoreductase1 C1 and C3 (AKR1C1 and AKR1C3). When AKR1C1 and AKR1C3 were inhibited, the combination of 3-MA paradoxically decreased the cell death rate of KATO-III induced by cisplatin [154].

Based on the data presented above using combinations of autophagy inhibitors with cisplatin, although most of the reported cisplatin-resistant cells demonstrated sensitization from the combination treatments, inefficient or even ineffective outcomes were also evident. Moreover, most of the experimental data were generated solely in in vitro cellular models. Furthermore, the paradigm that “autophagy is a drug resistance mechanism” may have occasionally resulted in a less than objective interpretations of the data.

In addition to these more classical autophagy inhibitors, there are some compounds and nanomaterials that have also been found to overcome cisplatin resistance by disrupting autophagy. U0126, a MAPK inhibitor, enhanced cisplatin-induced apoptosis by inhibiting autophagy in cisplatin-resistant ovarian cancer cells [155] and NSCLC cells [156]. Some natural products have also been found to promote the activity of cisplatin by regulating autophagy (Table 2). For example, astragaloside IV (AS-IV) derived from Astragalus membranaceus sensitizes cisplatin-resistant NSCLC cells to cisplatin by inhibiting ER stress and autophagy [157]. However, the role of autophagy induced by natural products in combination with cisplatin is not consistent. For instance, Gardenia jasminoides (GJ), a medicinal herb abundant with flavonoids, in combination with cisplatin paradoxically activated cytotoxic autophagy in glioblastoma multiforme [158]. In addition, the combination of autophagy-inhibiting nanoparticles or materials with cisplatin enhanced the chemosensitivity or reduced the resistance to cisplatin. For instance, a nanoparticle-based co-delivery system (siBec1@PPN) is centered on the efficient co-delivery of Beclin1 siRNA (Beclin1 is an autophagy initiation factor) and cisplatin to enhance the inhibitory effect on cisplatin-resistant A549 cells by inhibiting autophagy in vivo and in vitro [159]. Compared with the poly lactic acid (PLA) + cisplatin nanoparticles (CDDP-PLA NPs), the PLA + cisplatin-CQ nanoparticles (CDDP/CQ-PLA NPs) reduced autophagy and enhanced the ROS and apoptosis of Cal-27 cells [160].

One final issue that we suggest is worthy of additional attention is the “switch” between the roles played by autophagy in different tumors. As mentioned earlier, although in many if not most cases where cisplatin-induced autophagy has been detected the autophagy was functionally cytoprotective, particularly in cisplatin-resistant cells, the non-protective form of autophagy (i.e., where autophagy inhibition failed to influence cisplatin sensitivity) has also been observed, particularly in cisplatin-sensitive cells [50,77,100]. However, interestingly, re-expression of ARHI in SKOV3 cells allowed for CQ to suppress cisplatin sensitivity [77], whereas knockdown of p53 in H460 cells resulted in enhanced cisplatin sensitivity [50], which is what we refer to as the “autophagic switch”. This again argues that studies to improve the efficacy of chemotherapy by altering autophagy function should first distinguish the role of autophagy in specific models. In the absence of such a strategy, experimental conclusions in preclinical models may prove to be flawed, while translation of the work could be compromised.

### 3.2. Does Autophagy Inhibition Have the Potential to Exacerbate the Toxicity of Cisplatin to Normal Tissue?

The above extensive literature strongly suggests that inhibition of autophagy could, in fact, prove to be an effective strategy for combating cisplatin resistance in the clinic. However, this approach does not fully consider, for example, the troublesome issue of cisplatin toxicity to the kidneys. Cisplatin nephrotoxicity is related to the excretion of cisplatin, which occurs through the kidney tubular epithelial cells. Asymptomatic elevation of serum creatinine levels or even acute tubular injury requiring dialysis therapy occurs with cisplatin chemotherapy in the clinic. Patients who present with this condition often need to have their medication doses reduced to avoid further kidney damage, resulting in under-treatment of the disease [167]. Renal injury is often accompanied by other complications such as water and nitrogenous waste retention and is associated with a poorer patient prognosis [168]. Recent studies have shown that proximal tubule-specific autophagy-deficient mice are more susceptible to kidney injury after cisplatin treatment than wild-type mice [169]. This may be related to autophagy protecting the proximal tubular cells from mitochondrial oxidative stress and protecting the proximal tubular cells from DNA damage. Furthermore, autophagy also protects the proximal tubular cells from ischemic injury [170]. Zhang et al. reported that the mechanism of cisplatin nephrotoxicity may be related to the inhibition of autophagy by the activation of protein kinase C δ [171]. Li et al. found that 3-dehydroxyceanothetric acid 2-methyl ester (3DC2ME) isolated from the roots of jujube (*Ziziphus jujuba*, Rhamnaceae) protected against cisplatin-induced renal epithelial LLC-PK1 cell injury via autophagy modulation [172]. Retinoic acid, a major derivative of vitamin A, attenuates cisplatin-induced acute kidney injury by activating autophagy [173]. Numerous reports have demonstrated a protective effect of autophagy against cisplatin-induced renal cell injury [174]. Thus, prolonged coadministration of high doses of CQ or other autophagy inhibitors may exacerbate the nephrotoxicity of cisplatin. An example of this outcome can be taken from a study of amniotic fluid stem cells (AFSC) by Minocha et al., who found that AFSC reduced cisplatin-induced renal apoptosis in rats and served to protect against acute kidney injury, but CQ counteracted the renal protective effect of the AFSC [175]. The protective effect of autophagy was also demonstrated in cisplatin-induced damage to the cochlear cells [176]. In addition, the current study also demonstrates the importance of autophagy in enhancing the therapeutic potential of stem cell therapy in attenuating cisplatin-associated liver injury [177]. The two-sided nature of autophagy inhibition during cisplatin treatment suggests the necessity of elucidating the pattern of autophagy in therapy and finding ways to target the delivery of autophagy inhibitors to lesions while mitigating nephrotoxicity as well as other normal tissue injury associated with the administration of cisplatin in cancer therapy.

## 4. Autophagy in Cisplatin Combination with Immunotherapy

The Beth Levine laboratory reported 20 years ago that beclin 1+/− mice spontaneously develop lymphomas, lung cancer, as well as liver cancer [178,179], implicating autophagy in the protection of normal tissues from transformation. Subsequent studies found that this observation may be related to the role of autophagy in the immune system. Autophagy deficiency disrupts the clearance of malignant cells by dendritic cells and CD8+ T cells. Autophagy also has a facilitative role in tumor antigen cross-presentation and enhances the tumor responsiveness of the immune system [180]. However, autophagy can play an opposite role in the developmental process of established tumors. The deficiency of ATG7, an autophagy-associated gene that is essential for autophagosome production, delayed PTEN-deficient prostate tumor progression [181]. Lévy et al. showed that the loss of ATG7 promotes adaptive immunity, during which CD8+ T cells are necessary to prevent intestinal adenomas in APC+/− mice [182]. This suggests the need for autophagy as a “guardian” in the early stages of tumor formation and as an “accomplice” for tumor survival in the later stages.

During chemotherapy, autophagy also acts as a signal in the process of immune recognition and immunogenic cell death (ICD) [183,184]. Among the several cytotoxic antitumor compounds investigated (cisplatin, carboplatin, etoposide, paclitaxel, and gemcitabine), cisplatin was demonstrated to induce the highest levels of ICD-associated damage-associated molecular patterns (DAMPs) [185]. This study suggests the potential utility of cisplatin in combination with immunotherapy. A recent study showed that platinum-based chemotherapy synergizes with ErbB-targeted CAR-T cells, significantly reducing the tumor burden in mice, while co-treatment with the pharmacological autophagy inhibitor 3-MA caused a reversal in tumor cell suppression [186]. These results suggest a requirement for autophagy for the effectiveness of platinum-based chemotherapeutic agents, which argues against the strategy of autophagy inhibition.

Other studies, however, have suggested the necessity for autophagy inhibition in the utility of cisplatin with respect to the immune response. The tumor microenvironment is critical for chemotherapy efficacy where, for example, the polarization of tumor-associated macrophages (TAMs) influences tumor growth. Guo et al. found that the inhibition of autophagy using CQ would promote the polarization of tumor-associated macrophages to the M1 type, with an inhibitory effect on tumor proliferation and enhanced chemosensitivity of cisplatin [151]. LC3-associated phagocytosis (LAP) is a non-canonical autophagy that would be detected during phagocytosis by macrophages [187]. Recent studies have shown that blocking LAP inhibits the M2 type polarization of TAMs (a pro-tumoral proliferative state) and delays melanoma growth in vivo and in vitro [188]. These studies imply that the two-sided role of autophagy in the immune system is closely related to the timing of tumor development and furthermore that autophagy inhibition at a late stage of tumorigenesis might facilitate cisplatin activity.

## 5. Summary

Is autophagy always a barrier to cisplatin therapy? The currently available data indicate that the answer is nuanced and still uncertain. In general, the combination of autophagy inhibitors and cisplatin appears to have therapeutic potential for cisplatin-resistant tumors, but this approach likely cannot be generalized to cisplatin-sensitive tumors. With very few exceptions, the ongoing clinical trials of autophagy inhibition combined with cancer therapeutics have not yielded encouraging outcomes. Some fundamental reasons for this include that it has not been demonstrated conclusively that autophagy is consistently induced in malignancies by chemotherapy or radiation. Further, even if this were to be the case, there is no current approach that might indicate the nature of the induced autophagy. In addition, it is uncertain whether the tolerated dose of HCQ actually inhibits chemotherapy and radiation-induced autophagy and, if so, to what extent.

A clinical trial exploring the efficacy of combining CQ with cisplatin-etoposide in small-cell lung cancer (SCLC) patients was terminated 3 years after the initiation of the trial because of poor accrual (NCT00969306). Another clinical trial of CQ combined with cisplatin-etoposide for SCLC is currently ongoing in the Netherlands (EUCTR2009-014772-22-NL).

In addition to the reservations indicated above, how autophagy might influence the function of normal tissues such as the kidney (as indicated above), the GI tract where autophagy maintains integrity, or the central nervous system, wherein defective autophagy has been implicated in a number of pathological conditions, has generally not been given adequate consideration. Finally, the somewhat contradictory and still incomplete data relating to the influence of autophagy modulation on immune function with regard to tumor suppression indicates that we are not yet at a point where autophagy inhibition should be considered a viable clinical strategy in cancer therapeutics, whether in combination with cisplatin or other antitumor drugs or radiation.

## Figures and Tables

**Table 1:** Effect of CQ or HCQ on cisplatin-treated cancer cells.

Cancer Types	In Vitro Study Models	In Vivo Study Models	Effect of CQ or HCQ on Cisplatin Sensitivity	Reference
NSCLC	A549/cisplatin cells	-	Increased	[146]
	H460 cells	-	No effect	[50]
Endometrial cancer cells	Ishikawa/cisplatin cells	-	Increased	[147]
Urothelial carcinoma cells	RT-112/cisplatin cells	-	Increased	[148]
Ovarian cancer	A2780-CP20/cisplatin cells	An orthotopic mouse model established with A2780-CP20 cells and a drug-resistant patient-derived xenograft model	Increased	[149]
	ARHI-low expressed SKOV3 cells	-	No effect	[77]
Esophageal cancers	EC109/cisplatin cells	Nude mice xenografted with EC109/cisplatin cells	Increased	[29]
Neuroblastoma cells	Cisplatin-resistant model SK-N-BE(2)Cres cells	-	Increased	[150]
Oral squamous cell carcinoma	SCC-4 cells and SCC-4/cisplatin cells	-	Increased in SCC-4 cells, no effect in SCC-4/cisplatin cells	[152]
Pediatric medulloblastoma cells	DAOY and ONS76 cells	-	no effect	[153]
Breast cancer cells	67NR and 4T1 cells	-	no effect	[100]

**Table 2:** Role of autophagy in synergistic effects of natural products and cisplatin.

Compound	In Vitro Study Models	In Vivo Study Models	The Role of Autophagy in Cisplatin Only-Treated Models	Effect of Combination Treatment on Autophagy	Reference
Astragaloside IV (AS-IV) derived from Astragalus membranaceus	Cisplatin-resistant NSCLC cell lines	-	Unknown	Decreased autophagy levels	[157]
Hederagenin, a triterpenoid derived from Hedera helix	NSCLC cell lines NCI-H1299 and NCI-H1975	NCI-H1299 cells xenograft model	Unknown	Decreased autophagy levels	[161]
Acetyl-11-keto-β-boswellic acid (AKBA), a pentacyclic triterpenes, from Boswellia serrata	NSCLC cell lines A549	-	Unknown	Decreased autophagy levels	[162]
Andrographolide (Andro), one of the major active components in Andrographis paniculata	Cisplatin-resistant A549 cells	A549/cisplatin cells xenograft model	Unknown	Decreased autophagy levels	[163]
	Colon cancer cells HCT-116 (p53 wild type and p53-null)	-	Cytoprotective autophagy (both cell lines)	Decreased autophagy levels	[164]
Morin hydrate, a bioflavonoid, isolated from the Moraceae family	HepG2 cell	HepG2 xenograft nude mice	Unknown	Decreased autophagy levels	[165]
	Cisplatin-resistant HepG2 cells	Cisplatin-resistant HepG2 xenograft nude mice	Unknown	Decreased autophagy levels	[166]
Gardenia jasminoides (GJ) is a medicinal herb abundant with flavonoids	Glioblastoma multiform U87MG and U373MG cells	-	Unknown, but induced cytotoxic autophagy when combined with GJ	Increased cytotoxic autophagy levels	[158]

## Abbreviations

3-MA: 3-methyladenine; AMBRA1: activating molecule in Beclin1-regulated autophagy; APE1: apurinic/apyrimidinic endonuclease 1; ATG: autophagy-related; ATM: ataxia telangiectasia mutated protein; ATR: ataxia telangiectasia- and RAD3-related protein; CDKs: cyclin-dependent kinases; CQ: chloroquine; ECRG4: esophageal carcinoma-related gene 4; HCQ: hydroxychloroquine; MAP1S: microtubule-associated protein 1s; MDM2: murine double minute 2; MMR: mismatch repair; OPSCC: oropharyngeal squamous cell carcinoma; PFKFB3: 6-phosphofructo-2-kinase/fructose-2,6-biphosphatase 3 enzyme; RASSF1A: RAS association domain containing family 1A; ROS: reactive oxygen species; TAMs: tumor-associated macrophages.
